# Epigenetic Footprints of CRISPR/Cas9-Mediated Genome Editing in Plants

**DOI:** 10.3389/fpls.2019.01720

**Published:** 2020-01-31

**Authors:** Jun Hyung Lee, Mitra Mazarei, Alexander C. Pfotenhauer, Aubrey B. Dorrough, Magen R. Poindexter, Tarek Hewezi, Scott C. Lenaghan, David E. Graham, C. Neal Stewart

**Affiliations:** ^1^ Department of Plant Sciences, University of Tennessee, Knoxville, TN, United States; ^2^ Center for Agricultural Synthetic Biology, University of Tennessee Institute of Agriculture, Knoxville, TN, United States; ^3^ Department of Food Science, University of Tennessee, Knoxville, TN, United States; ^4^ Biosciences Division, Oak Ridge National Laboratory, Oak Ridge, TN, United States

**Keywords:** CRISPR/Cas9, genome editing, bisulfite sequencing, DNA methylation, epigenetic change

## Abstract

CRISPR/Cas9 has been widely applied to various plant species accelerating the pace of plant genome editing and precision breeding in crops. Unintended effects beyond off-target nucleotide mutations are still somewhat unexplored. We investigated the degree and patterns of epigenetic changes after gene editing. We examined changes in DNA methylation in genome-edited promoters of naturally hypermethylated genes (AT1G72350 and AT1G09970) and hypomethylated genes (AT3G17320 and AT5G28770) from *Arabidopsis*. Transgenic plants were developed via *Agrobacterium*-mediated floral dip transformation. Homozygous edited lines were selected from segregated T_2_ plants by an *in vitro* digestion assay using ribonucleoprotein complex. Bisulfite sequencing comparisons were made between paired groups of edited and non-edited plants to identify changes in DNA methylation of the targeted loci. We found that directed mutagenesis via CRISPR/Cas9 resulted in no unintended morphological or epigenetic alterations. Phenotypes of wild-type, transgenic empty vector, and transgenic edited plants were similar. Epigenetic profiles revealed that methylation patterns of promoter regions flanking target sequences were identical among wild-type, transgenic empty vector, and transgenic edited plants. There was no effect of mutation type on epigenetic status. We also evaluated off-target mutagenesis effects in the edited plants. Potential off-target sites containing up to 4-bp mismatch of each target were sequenced. No off-target mutations were detected in candidate sites. Our results showed that CRISPR/Cas9 did not leave an epigenetic footprint on either the immediate gene-edited DNA and flanking DNA or introduce off-target mutations.

## Introduction

As the global population continues to rapidly expand, food scarcity becomes a major issue. In recent decades, biotechnological advancements in genetic engineering have led to a great impact on modern agriculture through crop improvement. More recently, several precise genome editing tools have been developed using customizable, sequence-specific nucleases such as zinc-finger nucleases, transcription activator-like effector nucleases, and clustered regularly interspaced short palindromic repeats (CRISPR)/CRISPR-associated protein 9 (Cas9) (Gaj et al., 2013), fueling advances in the field of crop improvement. Among these genome editing approaches, CRISPR/Cas9 has rapidly become the best choice for gene editing in various plant species (reviewed by Jaganathan et al., 2018) because of its simplicity, efficiency, and design flexibility.

Despite its wide application for crop improvement, the risks of gene-editing technology need thorough examination. Off-target effects are one of the major concerns of using CRISPR/Cas9 because Cas9 is tolerant to some mismatched sequences distal from the protospacer adjacent motif (PAM). Although the off-target effects are unlikely to occur in plants (Bao et al., 2019), high-frequency off-target mutagenesis was reported in human cells (Fu et al., 2013) and represents a potential limitation for biomedical and clinical applications (Zhang et al., 2015). Therefore, many studies have been conducted to minimize off-target effects and to improve specificity of CRISPR/Cas9 (reviewed by Moon et al., 2019). Unintended effects beyond off-target mutations, however, are still somewhat unexplored. We know of no published studies that have explored epigenetic changes in genome-edited organisms.

Epigenetic changes, such as DNA methylation, histone modifications, and non-coding RNA changes, can affect genome stability and gene expression. DNA methylation is the most common epigenetic ‘footprint’ in plants and plays important roles in various biological processes such as transposon silencing, plant development, and plant responses to biotic and abiotic environmental stimuli (reviewed by Zhang et al., 2018). The patterns of methylation are important in various regions of DNA, including promoters. For example, genome-wide mapping of DNA methylation in *Arabidopsis* showed that, in general, methylation in transcribed regions tends to promote higher and constitutive gene expression, while genes methylated in promoter regions show tissue-specific expression (Zhang et al. 2006b). Promoter DNA methylation is usually associated with gene repression by direct inhibition of the binding of transcription factors (Domcke et al., 2015; Zhu et al., 2016), but in some cases it promotes gene expression by a still unknown mechanism (Song et al., 2013; Lang et al., 2017).

In the present study, we aim to assess epigenetic changes attributable to CRISPR/Cas9-mediated genome editing in plants. To evaluate epigenetic profiles, especially with regard to promoter methylation, we selected four genes in which a differentially methylated region was located on their promoter: naturally hypermethylated genes (AT1G72350 and AT1G09970) and hypomethylated genes (AT3G17320 and AT5G28770) from *Arabidopsis*. To our knowledge, this is the first study about potential effects of genome editing on DNA methylation status in plants.

## Materials and Methods

### Selection of CRISPR/Cas9 Target Sites and Designing gRNAs

Four *Arabidopsis* genes (AT1G72350, AT1G09970, AT3G17320, and AT5G28770) containing differentially methylated regions (200-bp long) in their promoters (Hewezi et al., 2017) were selected for CRISPR/Cas9-mediated genome editing. The 200-bp sequence was entered in the web-based tool CRISPOR (http://crispor.tefor.net/crispor.py; Haeussler et al., 2016) to design optimal gRNAs for each target in consideration of their GC content and the presence of potential off-target sites (**Table 1**). Polymerase chain reaction (PCR) products flanking the target sites were sequenced to evaluate any possible allelic variation or single-nucleotide polymorphisms. Unless noted otherwise, Phusion High-Fidelity DNA polymerase (New England Biolabs, Ipswich, MA) was used for all PCR. The primer sequences used in this study are listed in **Supplementary Table S1**.

**Table 1:** Target genes containing differentially methylated regions (200-bp) in their respective promoters, selected gRNA sequences within these regions, and gRNA GC-contents.

Gene ID	Differentially methylated region	gRNA sequence^z^	gRNA GC-content
AT1G72350	Chr1: 27240601-27240800	AATACTGACTAATGAACCCG*TGG*	40%
AT1G09970	Chr1: 3251401-3251600	CTACACTACATGGTAGGCTT*AGG*	45%
AT3G17320	Chr3: 5917401-5917600	TTAAAGGTGGTACCAGCAGT*TGG*	45%
AT5G28770	Chr5: 10798801-10799000	CCCTTTATGGTAGAGGACGT*TGG*	50%

^z^Protospacer adjacent motif (PAM) is indicated in italics.

### Vector Construction

The binary vector pKSE401 (Addgene plasmid # 62202; Xing et al., 2014) was used in this study to generate sgRNA/Cas9 constructs. The synthesized oligonucleotides of gRNAs (Integrated DNA Technologies, Coralville, IA) specific to the target sequence with appropriate overhangs were annealed and then ligated into the BsaI site in the pKSE401 vector. In brief, two complementary single stranded oligonucleotides (one strand consisting of a 5ˊ-ATTG overhang plus 20-nt gRNA sequence, and the other strand consisting of a 5ˊ-AAAC overhang and the complementary sequence) were mixed together and incubated at 95°C for 2 min followed by cooling to 4°C using a thermal cycler. The pKSE401 vector was digested by BsaI, then the annealed double strand fragments were ligated into the vector.

### Generation of Stable Transgenic Lines

The pKSE401 vectors harboring the sgRNA/Cas9 expression cassette were transformed into *Agrobacterium tumefaciens* strain EHA105 using the freeze-thaw method. For an empty vector control, pKSE401 vector without any gRNA sequence was transformed. The binary vectors were then introduced into *Arabidopsis thaliana* Col-0 plants via the floral dip method (Zhang et al. 2006a). T_1_ seedlings were selected on Murashige and Skoog plates containing kanamycin (50 mg/l) for 2 weeks, and then transplanted to soil for further growth for genotyping and selfing to the next generation. For bisulfite sequencing, homozygous edited plants were selected in the T_2_ generation. Experimental plants were grown in a growth room kept at 23°C under a 16 h photoperiod.

### Genotyping and Detection of Mutation Using PCR/Ribonucleoprotein (RNP) Complex

Genomic DNA from T_1_ plants was extracted using the CTAB method and the presence of the *Cas9* gene was confirmed by PCR. PCR amplifications were carried out with an initial denaturation step at 98°C for 30 s, followed by 35 cycles comprising of 98°C for 10 s, 60°C for 30 s, and 72°C for 20 s, and a final extension for 10 min at 72°C. From the PCR-confirmed transgenic lines, DNA fragments flanking the target sites were amplified using specific primers and then directly sequenced by Sanger method to find edited lines. To select homozygous edited plants in the T_2_ generation, a genotyping method using PCR/RNP complex was adopted (Liang et al., 2018). In brief, template DNAs were amplified using specific primers and then used for *in vitro* sgRNA synthesis using HiScribe^TM^ T7 Quick High Yield RNA Synthesis Kit (New England Biolabs) according to the manufacturer’s instructions. sgRNAs were then cleaned by Monarch^®^ RNA Cleanup Kit (New England Biolabs) before adding to the Cas9 digestion mixture. For each digestion reaction, RNP complex in Cas9 reaction buffer (100 mM NaCl, 50 mM Tris-HCl, 10 mM MgCl_2_, 100 µg/ml BSA) consisted of 0.5 µg sgRNA, 0.5 µg Cas9 protein, and ddH_2_O up to 10 µl. The mixture was pre-incubated at 25°C for 10 min before adding 1 µl of PCR amplicon flanking the target site. The PCR/RNP mixture was then incubated at 37°C for 3 h for digestion, followed by incubation at 65°C for 10 min to stop the reaction. The reaction products were then analyzed immediately on 2% agarose gel. Only the PCR products undigested by RNP, which were assumed as homologous or biallelic mutants, were selected for direct sequencing to determine mutations.

### Bisulfite Genomic Sequencing

To avoid variability caused by inbreeding, T_2_ homozygous plants from segregating populations were used for DNA methylation experiments. Experiments consisted of up to four independent biological replicates for each construct. Genomic DNA was extracted from leaves of 4-week-old plants using the Plant DNeasy Mini Kit (Qiagen, Germantown, MD). Aliquots of 500 ng of each DNA sample were subjected to bisulfite treatment using the EZ DNA Methylation-Gold Kit (Zymo Research, Irvine, CA) according to the manufacturer’s protocol. PCR was performed by using bisulfite-treated DNA as templates. The sequences of interest were amplified using polymerase Takara Ex Taq Hot Start (Clontech, Mountain View, CA) and methylation-neutral primers. PCR amplifications were carried out with an initial denaturation step at 94°C for 5 min, followed by 40 cycles comprising of 94°C for 45 s, 55°C for 45 s, and 72°C for 1 min, and a final extension for 10 min at 72°C. The PCR-amplified fragment of each bisulfite-treated DNA sample was gel-purified using the Zymoclean Gel DNA Recovery Kit (Zymo Research) and cloned into the pGEM-T Easy vector (Promega, Madison, WI). For each PCR amplicon, at least seven independent colonies were analyzed by Sanger sequencing. Unmethylated lambda phage DNA (Promega) was used as a control for bisulfite conversion efficiency. The primers used in this study are listed in **Supplementary Table S1**.

### Off-Target Analysis

The potential off-target sites containing up to 4-bp mismatches were predicted by the web-based tool CRISPOR. Genomic DNA was extracted from selected T_2_ plants and PCR was performed using primer sets designed to flank the potential off-target sites. PCR products were then gel-purified using Zymoclean Gel DNA Recovery Kit (Zymo Research) and directly sequenced to evaluate off-target mutagenesis.

## Results

### CRISPR/Cas9 Targets and gRNA Design

**Figure 1:**
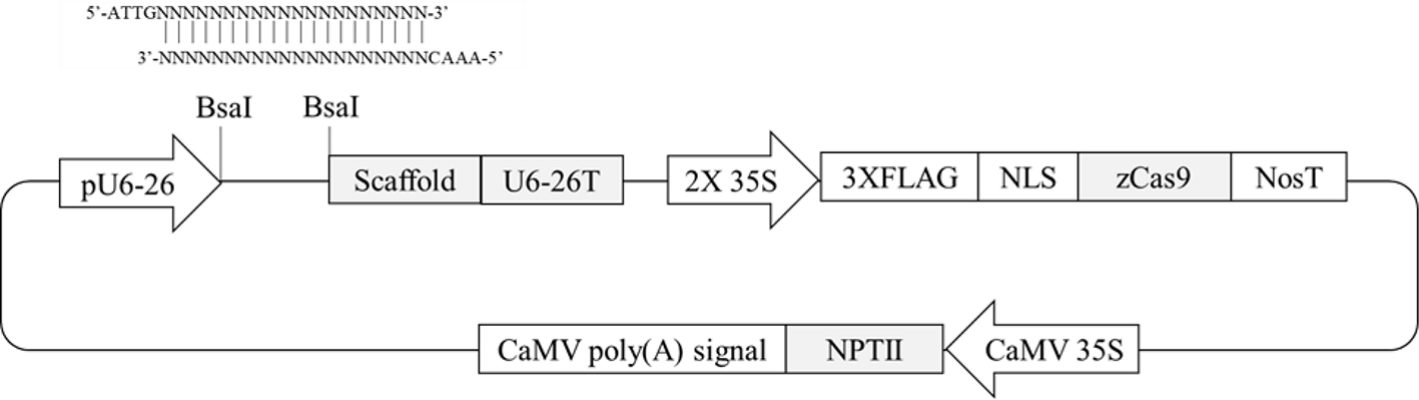
Schematic diagram of CRISPR/Cas9 plant transformation vector pKSE401. Oligonucleotides of gRNA sequence were annealed and then inserted at the BsaI-digestion site between the pU6-26 promoter and gRNA scaffold.

Four *Arabidopsis* genes containing differentially methylated regions (DMRs) in their promoters (Hewezi et al., 2017) were selected. Two hundred-bp promoter regions of two genes (AT1G72350 and AT1G09970) are naturally hypermethylated, whereas promoters of the other two genes (AT3G17320 and AT5G28770) are hypomethylated (Hewezi et al., 2017). CRISPR/Cas9 target sites were selected within the DMRs. To evaluate any possible allelic variation or single-nucleotide polymorphisms in the DMRs, genomic DNA was extracted from randomly selected wild-type plants, and the PCR amplicons flanking the regions were directly sequenced. The results confirmed that there were no variations within the selected target sites (data not shown). To make CRISPR/Cas9 vectors, two complementary oligonucleotides of gRNA sequence were annealed together and then placed between AtU6-26 promoter and gRNA scaffold sequence in pKSE401 vector in which *Zea mays* codon-optimized *Cas9* was expressed by a CaMV 35S promoter (**Figure 1**). A total of four vectors were constructed to target the four genes to study the effects of genome editing on DNA methylation status.

### CRISPR/Cas9-Mediated Mutagenesis

CRISPR/Cas9 vectors were transformed into *A. thaliana* Col-0 plants via the floral dip method, and putative transgenic plants were screened by kanamycin selection. Six to fourteen T_1_ plants per construct, in which the presence of Cas9 vector was confirmed by PCR, were used for genotyping to detect mutation and selfing to the next generation. PCR amplicons flanking the target sites were directly sequenced to check for mutations. The mutagenesis rates in T_1_ plants varied with a range of 30% to 100% depending on the target genes, but all the edited plants were heterozygous or biallelic mutants with multiple peaks on the sequence chromatogram downstream from the expected Cas9 cleavage sites (**Table 2**; **Supplementary Figures S1**–**S4**).

**Table 2:** Mutation events in T_1_ transgenic lines of the target genes.

Gene ID	No. examined T_1_ lines	Zygosity			Mutation rate
		Homozygote	Heterozygote/Biallele	Wild-type
AT1G72350	10	0	3	7	30.0%
AT1G09970	6	0	6	0	100.0%
AT3G17320	12	0	7	5	58.3%
AT5G28770	7	0	6	1	85.7%

**Figure 2:**
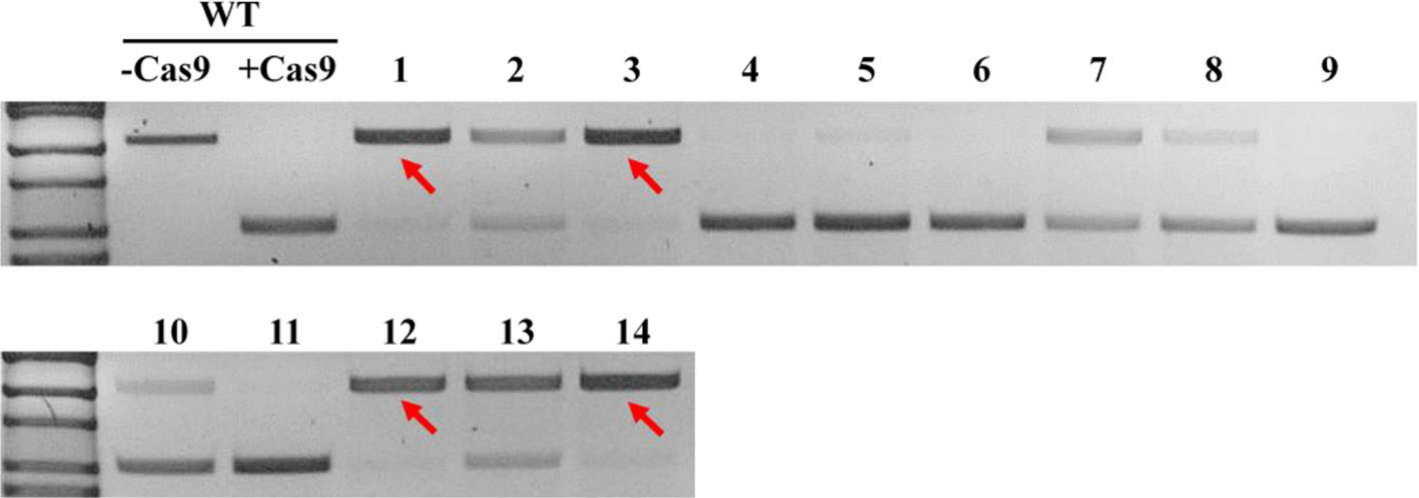
Representative genotyping result using PCR/ribonucleoprotein (RNP) complex to select potential homozygous lines. Independent edited plants in the T_2_ generation targeting AT1G72350 were analyzed using PCR/RNP complex, a mixture of *in vitro* synthesized sgRNA, Cas9 enzyme, and PCR products flanking target site. PCR amplicons identical to the sgRNA were completely digested by Cas9 (lanes 4, 5, 6, 9, and 11), while amplicons of homozygous or biallelic-mutants (red arrows) were not digested. Partially digested amplicons showing both cut- and uncut-bands indicated heterozygous mutants (lanes 2, 7, 8, 10, and 13).

**Figure 3:**
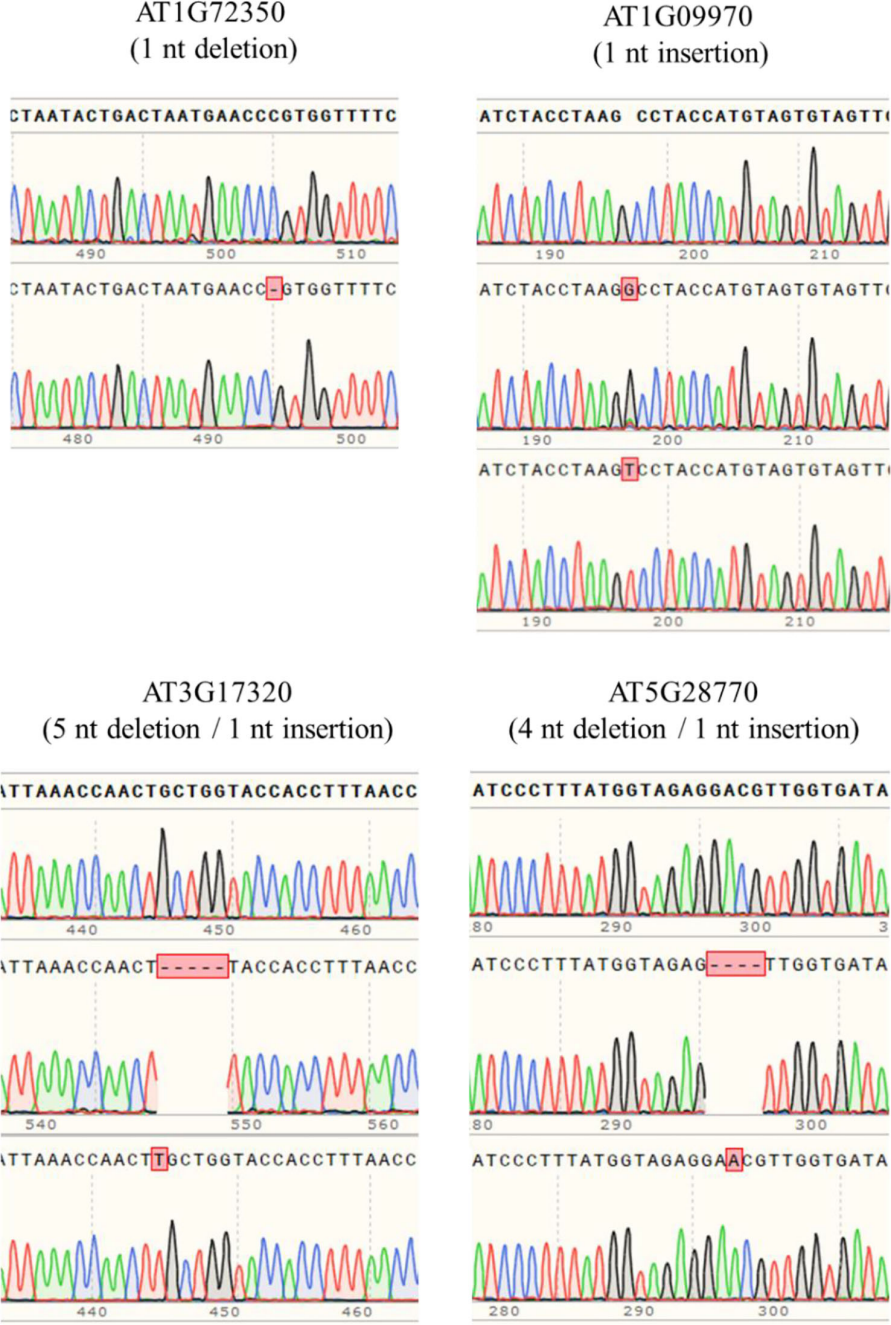
Detection of mutations by the Sanger sequencing method. PCR products flanking target sites were amplified from putative homozygous edited T_2_ plants, and then directly sequenced. Sequence alignment showed that 1-nt insertion/deletion was the major mutation type, but up to 5-nt deletion occurred.

**Figure 4:**
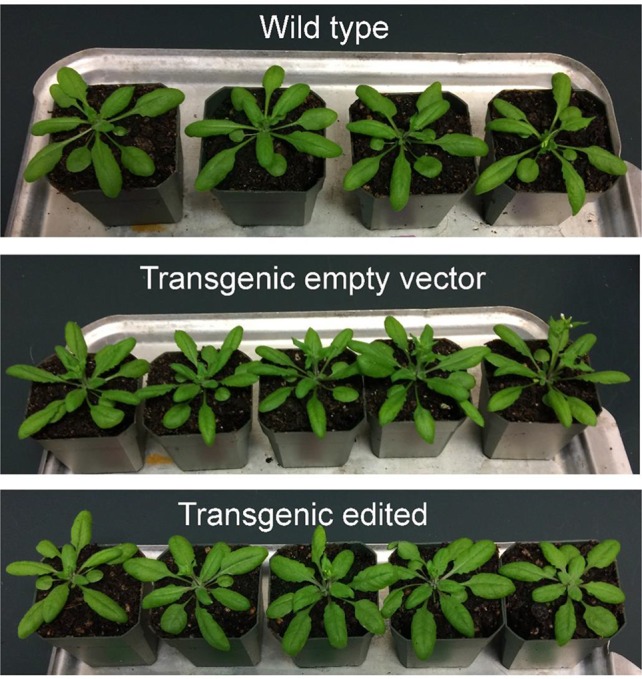
Representative wild type, transgenic empty vector, and transgenic edited T_2_ plants at 4 weeks old.

In order to obtain homozygous edited plants for bisulfite sequencing, T_2_ plants were generated. To minimize the number of plants required for sequence confirmation from approximately twenty T_2_ seedlings per edited line, a recently developed genotyping method using PCR/RNP complex was applied (Liang et al., 2018). Because homozygous or biallelic mutants have lost their complementary sequence to the gRNA, PCR amplicons of the target site are not digested by Cas9, resulting in a single uncut band only (**Figure 2**; lanes 1, 3, 12, and 14). PCR amplicons from heterozygous mutants, however, contained both edited and original gRNA sequences that were partially digested by Cas9, which resulted in two bands (**Figure 2**; lanes 2, 7, 8 10, and 13). Unedited T_2_ plants were also generated by segregation from heterozygous T_1_ mutants, and their PCR amplicons were completely digested like the wild-type control (**Figure 2**; lanes 4, 5, 6, 9, and 11). Based on the digestion pattern from the PCR/RNP assay, we selected the lines with exclusively uncut bands to be used for further sequence analysis. Direct sequencing of the PCR amplicons detected mutations for all four target genes: 1-nt deletion (C) in AT1G72350; 1-nt insertion (+G or +T) in AT1G09970; 1-nt insertion (+T) and 5-nt deletion (GCTGG) in AT3G17320; and 1-nt insertion (+A) and 4-nt deletion (GACG) in AT5G28770 (**Figure 3**). No phenotypic differences were apparent (**Figure 4**), and we used these plants for allele-specific bisulfite sequencing analysis.

### Off-Target Analysis

According to analysis using the web-based tool CRISPOR, there were no homologous sequences to the four gRNAs flanking a PAM sequence, and no loci containing even up to 3-nt mismatches were found. Potential off-target sites with 4-nt mismatches were predicted for three target genes: 8 loci for AT1G72350; 2 loci for AT1G09970; and 6 loci for AT3G17320 (**Table 3**). The potential off-target sites were amplified from selected T_2_ plants using the specific primers and then directly sequenced. Sequence alignment analysis revealed that no off-target mutagenesis occurred. We also checked the presence of CRISPR/Cas9 construct in the selected T_2_ plants. Most of the plants still contained the construct (**Supplementary Figure S5**) with the exception of three lines targeting AT3G7320, indicating off-target mutagenesis on loci with 4-nt mismatches may not be likely to occur in plants by CRISPR/Cas9 editing.

**Table 3:** Potential off-target analysis of edited T_2_ plants.

Target	Putative off-target locus	Putative off-target sequence^z^	No. mismatch bases	No. mutations
AT1G72350	Chr1: 6819675-6819745	AATACGCACTAATGAACCTT*TGG*	4	0
	Chr5: 19687368-19687402	*CCA*CGGGTTAATTAGTTAAAATT	4	0
	Chr1: 6004797-6004831	AATATTGACTAAGGAACTTG*AGG*	4	0
	Chr5: 18500808-18500842	*CCA*CGGGTTAATTAGTTAAAATT	4	0
	Chr3: 18059211-18059233	*CCA*CGGGTTAATTAGTTAAAATT	4	0
	Chr3: 18055348-18055370	*CCA*CGGGTTAATTAGTTAAAATT	4	0
	Chr1: 22778972-22778994	AATTTTAACTAATTAACCCG*TGG*	4	0
	Chr1: 22779505-22776527	AATTTTAACTAATTAACCCG*TGG*	4	0
AT1G09970	Chr5: 23029175-23029217	CGACAATACTTGATAGGCTT*TGG*	4	0
	Chr5: 1736941-1736983	*CCA*AAGCATACTATGCAGTGGAG	4	0
AT3G17320	Chr5: 21349929-21349971	TTAAAGGTGTTACTAACAAT*GGG*	4	0
	Chr1: 20736302-20736344	TTAAAAGTGAAACCAGCTGT*GGG*	4	0
	Chr5: 577830-577872	TTGAAAGTGGTACCAGCTGA*TGG*	4	0
	Chr5: 477720-477762	*CCA*TATGCTGGGATCACCTTTAA	4	0
	Chr2: 2191921-2191963	*CCT*CCTGCTGGTACAAACTTTGA	4	0
	Chr1: 10628995-10629038	*CCT*ACTACTGTAACCACCATTA	4	0

^z^Mismatch nucleotides are marked in red and protospacer adjacent motif (PAM) is indicated in italics.

### DNA Methylation

**Figure 5:**
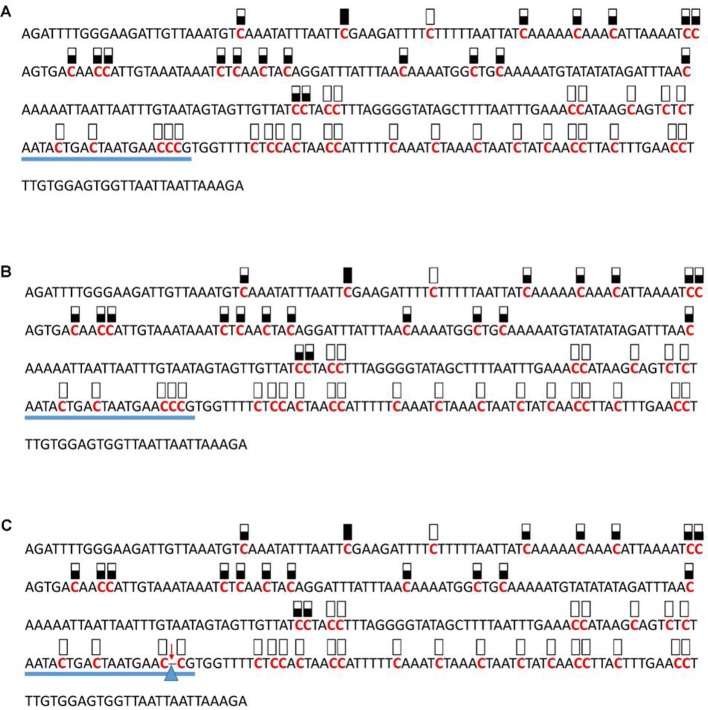
Pattern of methylation of the AT1G72350 promoter in wild-type **(A)**, transgenic empty vector **(B)**, and transgenic edited **(C)** plants. Because the data were obtained by sequencing of independent pGEM-T colonies, an average level of methylation was determined for each cytosine. Solid boxes indicate that the cytosine at this position were methylated (>90%), open boxes indicate that cytosine methylation was not detected (<10%), and half-shaded boxes indicate that the cytosine was methylated at a range of 40-60%. Underlines indicate the 20-bp target sequences. The arrow and triangle in **(C)** indicate the one nucleotide “C” deletion. This figure presents results of 4 independent biological replicates.

**Figure 6:**
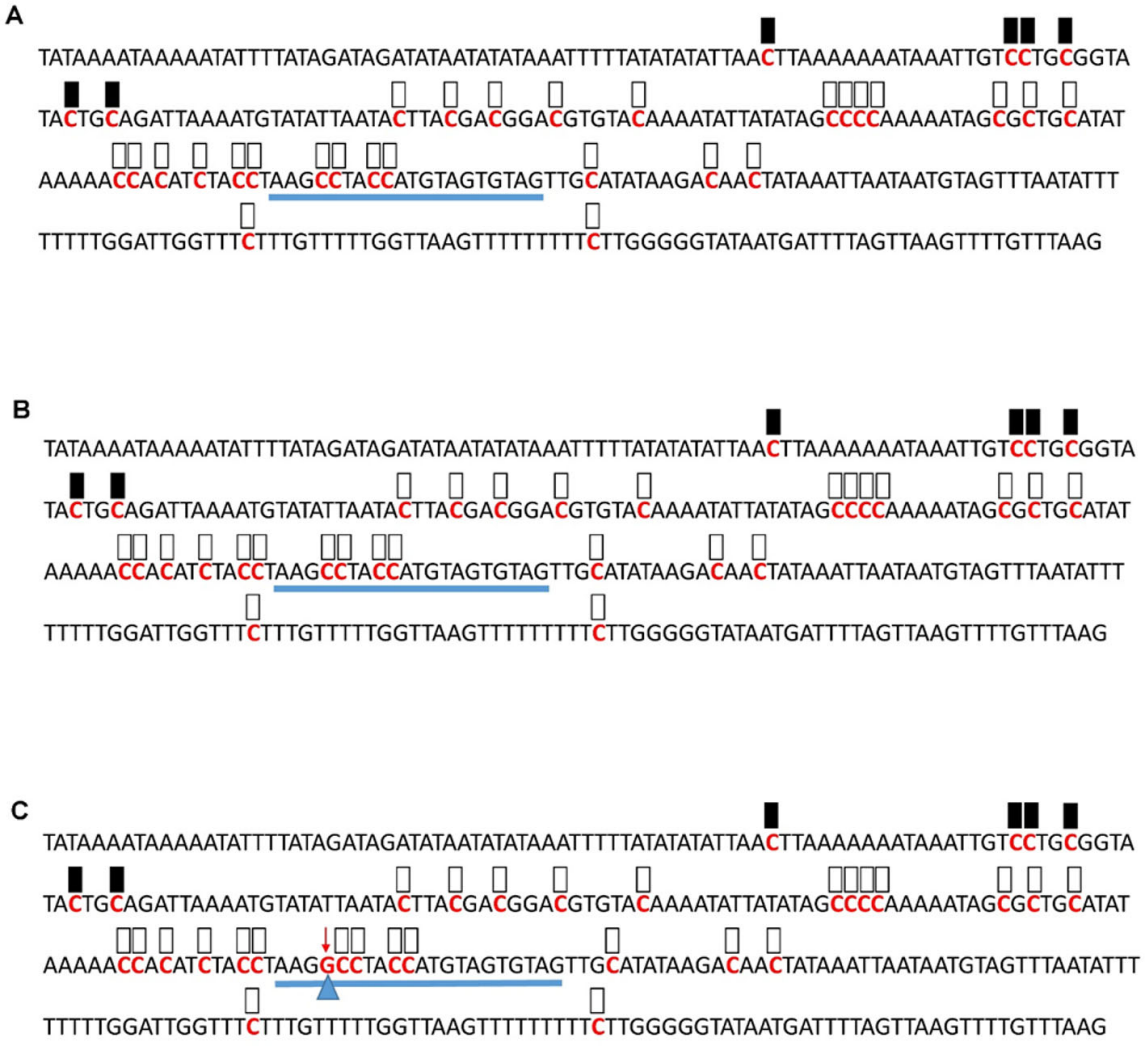
Pattern of methylation of the AT1G09970 promoter in wild-type **(A)**, transgenic empty vector **(B)**, and transgenic edited **(C)** plants. Because the data were obtained by sequencing of independent pGEM-T colonies, an average level of methylation was determined for each cytosine. Solid boxes indicate that the cytosine at this position were methylated (>90%), open boxes indicate that cytosine methylation was not detected (<10%), and half-shaded boxes indicate that the cytosine was methylated at a range of 40-60%. Underlines indicate the 20-bp target sequences. The arrow and triangle in **(C)** indicate the one nucleotide “G” addition. This figure presents results of 4 independent biological replicates.

**Figure 7:**
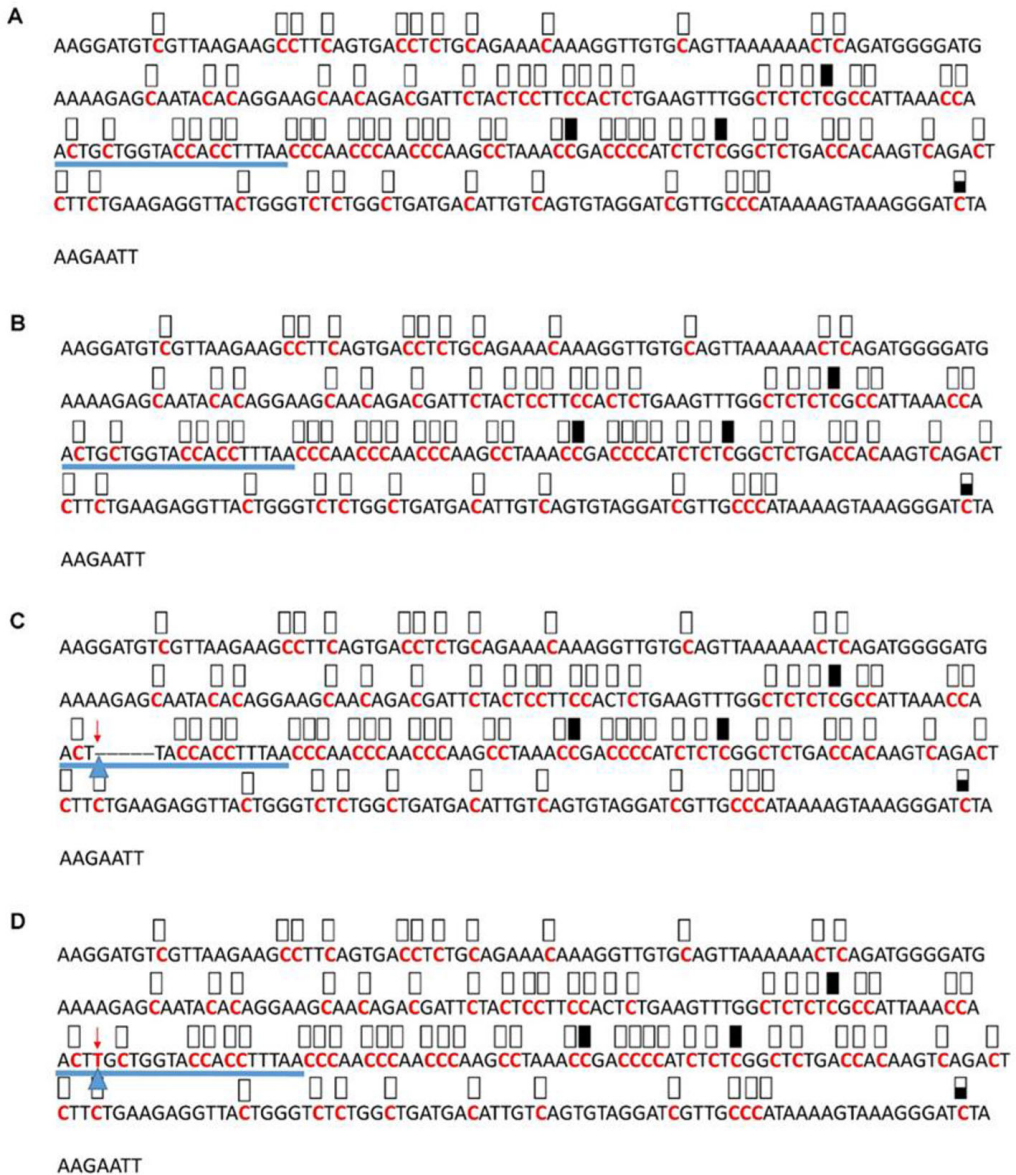
Pattern of methylation of the AT3G17320 promoter in wild-type **(A)**, transgenic empty vector **(B)**, and transgenic edited **(C**, **D)** plants. Because the data were obtained by sequencing of independent pGEM-T colonies, an average level of methylation was determined for each cytosine. Solid boxes indicate that the cytosine at this position were methylated (>90%), open boxes indicate that cytosine methylation was not detected (<10%), and half-shaded boxes indicate that the cytosine was methylated at a range of 40-60%. Underlines indicate the 20-bp target sequences. The arrow and triangle in **(C)** indicate the five nucleotide “GCTGG” deletion. The arrow and triangle in **(D)** indicate the one nucleotide “T” addition. This figure presents results of 4 independent biological replicates.

**Figure 8:**
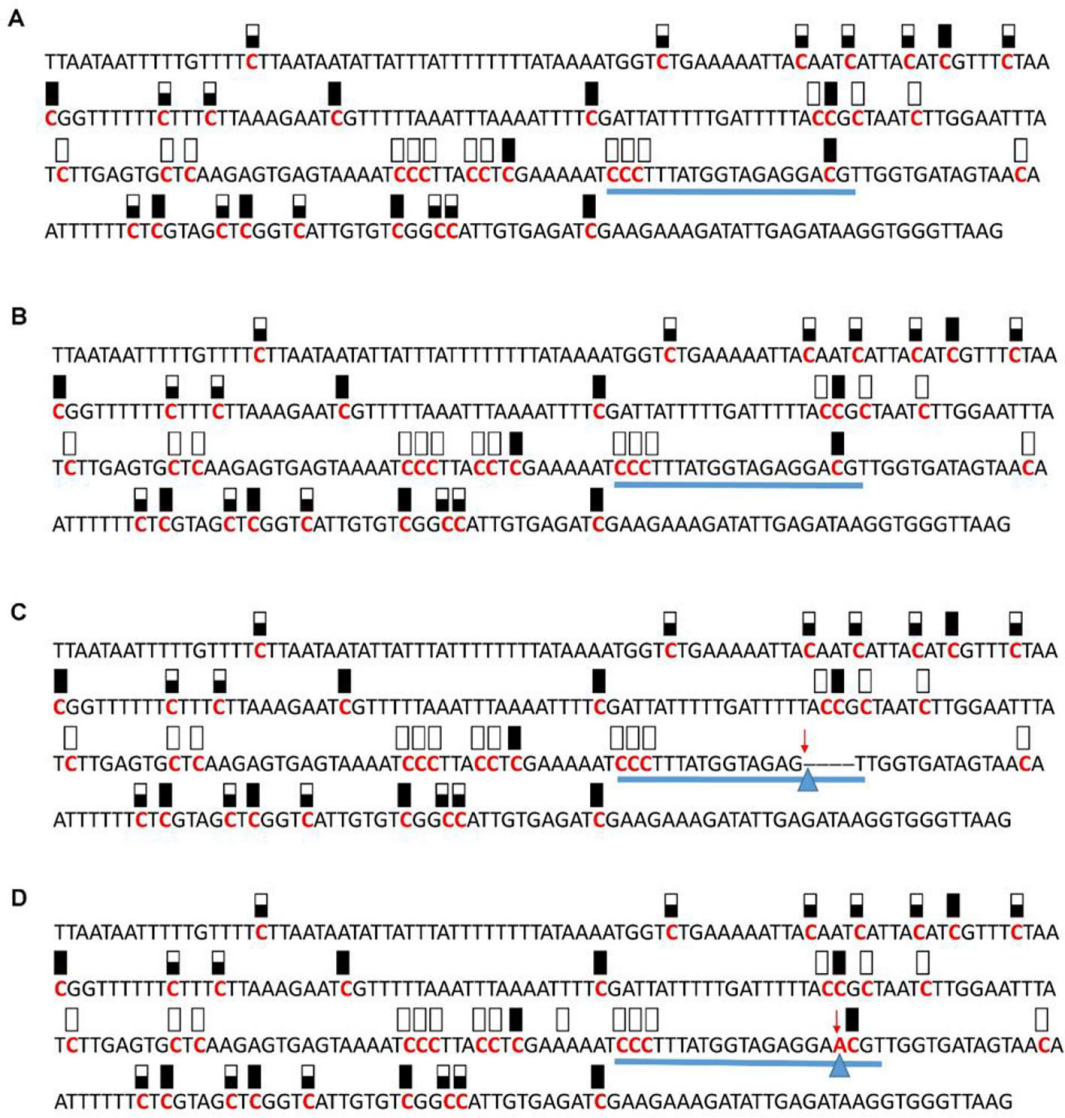
Pattern of methylation of the AT5G28770 promoter in wild-type **(A)**, transgenic empty vector **(B)**, and transgenic edited **(C**, **D)** plants. Because the data were obtained by sequencing of independent pGEM-T colonies, an average level of methylation was determined for each cytosine. Solid boxes indicate that the cytosine at this position were methylated (>90%), open boxes indicate that cytosine methylation was not detected (<10%), and half-shaded boxes indicate that the cytosine was methylated at a range of 40-60%. Underlines indicate the 20-bp target sequences. The arrow and triangle in **(C)** indicate the four nucleotide “GACG” deletion. The arrow and triangle in **(D)** indicate the one nucleotide “A” addition. This figure presents results of 4 independent biological replicates.

To identify changes in DNA methylation patterns associated with CRISPR/Cas9-mediated genome editing, we used the bisulfite sequencing approach for analyzing bisulfite-converted DNA providing single-base resolution across the entire amplicon. Experiments were performed with wild-type, transgenic empty vector, and transgenic edited plants (**Figure 4**). Cloning of bisulfite PCR products followed by sequencing with vector-specific primers was performed to obtain the best sequencing results for quantification of methylation. The degree of bisulfite-conversion was determined by sequencing. Bisulfite conversion efficiency was up to 97%, as determined using unmethylated lambda phage DNA. DNA methylation of the edited plants in the locus-specific gene-edited promoters of hypermethylated (AT1G72350 and AT1G09970) and hypomethylated (AT3G17320 and AT5G28770) genes was compared to that of the control plants. These analyses showed no alterations in methylation patterns of the corresponding locus in wild-type, transgenic empty vector, and transgenic edited plants (**Figures 5**–**8**). Also, there was no apparent association between types of mutation and methylation patterns (**Figures 7C**, **D** and **8C**, **D**). We also performed cross-check analysis to see if there were methylation pattern changes in other areas of the genome that was not targeted: (AT1G72350) region in AT1G09970-edited plant; (AT1G09970) region in AT1G72350-edited plant; (AT3G17320) region in AT5G28770-edited plant; and (AT5G28770) region in AT3G17320-edited plant. We selected these genes since in the previous study by Hewezi et al., 2017, these genes showed differentially methylated patterns by changing the methylation status in response to stimuli. We wanted to examine whether Cas9-associated biological activities affect DNA methylation status not just on the target sites where Cas9 binding followed by DNA repair processes, but also on other sites in the genome. No differences in methylation patterns of the target regions were detected between the wild type and the edited plants (**Supplementary Figures S6**–**S9**). Altogether, our results suggest that CRISPR/Cas9-mediated genome editing did not cause unintended effects and did not leave epigenetic artifacts on target sequences.

## Discussion

CRISPR/Cas9 approaches are powerful tools for crop improvement and are largely considered to be viable precision-breeding methods (Scheben et al., 2017). For example, major traits associated with productivity in ‘groundcherry’ (*Physalis pruinsa*) were improved by CRISPR/Cas9-mediated mutation on orthologues of tomato domestication and improvement genes, *SELF-PRUNING* genes, and *CLAVATA1* gene that control plant architecture, flower production, and fruit size (Lemmon et al., 2018). Although some CRISPR/Cas9-edited crops are not currently regulated as genetically modified organisms (GMO) by the USDA in the United States (Waltz, 2018), the controversy surrounding the regulation of genome-edited crops is still in flux. The Court of Justice of the European Union (ECJ) recently ruled that CRISPR/Cas9-edited plants should be subject to the same draconian GM regulations in the EU (Callaway, 2018). In order to answer regulatory questions and engender public acceptance, potential adverse effects in genome-edited plants should be thoroughly assessed. In the present study, we provided locus-specific epigenetic profiles of edited plants to assess potential adverse effects of plant genome editing by CRISPR/Cas9. We generated transgenic *Arabidopsis* plants to edit four target genes that have differentially methylated regions in their promoters using CRISPR/Cas9. In T_1_ plants, we obtained 30—100% mutation frequencies (**Table 2**), which were similar to published studies. In dicot plants, a wide range of mutation frequency has been reported in CRISPR/Cas9-mediated genome editing, ranging from 30% to 92% in *Arabidopsis* (Feng et al., 2014); from 81.8% to 87.5% in tobacco (Gao et al., 2015); from 84% to 100% in tomato (Pan et al., 2016); and from 20% to 100% in soybean (Cai et al., 2018). Our previous study suggested that gRNA GC-content may play a role in sgRNA efficacy; no edited plants were generated when gRNAs with less than 40% GC-content were used, and a higher mutation frequency was obtained when a higher GC-content was used (unpublished data). Similar results were reported in other studies (Zhang et al., 2014; Pan et al., 2016), but very high GC-content was also less effective and may increase the risk of off-target cleavage (Wang et al., 2014; Tsai et al., 2015).

Off-target mutation is a common and critical problem associated with CRISPR/Cas9-mediated genome editing in human cells, but it has rarely been reported in gene-edited plants. Several studies have reported that there was no off-target mutation, not only in *Arabidopsis*, but also various crop species (Feng et al., 2014; Pan et al., 2016; Braatz et al., 2017; Jia et al., 2017; Tang et al., 2017; Cai et al., 2018). We analyzed the potential off-target mutagenesis on loci highly similar to the target sequence. No mutations were detected at any of these candidate off-target sites (**Table 3**), demonstrating that we designed highly specific gRNAs. It has been proposed that the risk of off-target mutations in plants, unlike that in therapeutic applications, may not be a critical issue because any unwanted phenotypic effects caused by off-target mutations or somatic mutations can be eliminated by the subsequent selection process of plant tissue culture-based transformation after editing (Zhao and Wolt, 2017).

DNA methylation is one of the most extensively studied epigenetic modifications of genomic DNA. Numerous DNA methylation studies have demonstrated that DNA methylation is critical in many regulatory processes such as silencing of gene expression, cellular differentiation, transposon mobility, genome stability, and genomic imprinting (Bewick and Schmitz, 2017; Elhamamsy, 2017; Niederhuth and Schmitz, 2017; Yaish, 2017; Bartels et al., 2018; Bräutigam and Cronk, 2018; Zhang et al., 2018). Much effort has been paid to characterize variation of DNA methylation across different biological samples, developmental stages, and disease status (Zhang et al. 2006b; Dinh et al., 2012; Breuil-Broyer et al., 2016; Hewezi et al., 2018; Xu et al., 2018), however, changes in DNA methylation patterns associated with CRISPR/Cas9-mediated genome editing have not been explored yet. We examined the impact of CRISPR/Cas9-mediated genome editing on the DNA methylation patterns of four gene promoters (hypermethylated genes AT1G72350 and AT1G09970; and hypomethylated genes AT3G17320 and AT5G28770) in which differentially methylated region was located on their promoter (Hewezi et al., 2017). We conducted targeted DNA methylation analysis by treating genomic DNA with bisulfite, amplifying and sequencing targeted regions of interest. Bisulfite genomic sequencing is regarded as a gold-standard technology for detection of DNA methylation over the genomes of interest because it provides a qualitative, quantitative, and efficient approach to identify methylcytosine at single base-pair resolution (Li and Tollefsbol, 2011; Wreczycka et al., 2017). Bisulfite-mediated cytosine conversion paired with subsequent PCR amplification and sequencing represents a highly promising approach (Henderson et al, 2010; Masser et al., 2015). We performed Sanger sequencing of bisulfite converted DNA, which is the most used methods for analysis of targeted regions (i.e., a promoter region of a single gene). Under these experimental conditions, our results showed that CRISPR/Cas9-mediated genome editing did not change DNA methylation patterns among wild-type, transgenic empty vector, and transgenic edited plants, regardless of the types of mutation caused by CRISPR/Cas9-mediated gene editing in the same target gene promoter and/or between the gene promoters by cross-check analysis. Our results suggest that there was not an association between CRISPR/Cas9-mediated genome editing and DNA methylation. In this context, given the increased knowledge about highly efficient CRISPR/Cas9 as the most-used genome editing tool, determining unintended effects beyond off-target mutations such as methylation status presented here provides further insights into the application of this precise genome editing platform.

## Data Availability Statement

All datasets generated for this study are included in the article/**Supplementary Material**.

## Author Contributions

JL and MM designed and performed the experiments, analyzed the data, and wrote the manuscript. AP designed gRNAs and performed cloning of CRISPR/Cas9 constructs. AD conducted off-target analysis. MP performed genomic DNA extractions, PCR, cloning of bisulfite converted fragments, and assisted with plant care. TH, SL, DG, and CS conceived of the study and its design and coordination, and assisted with interpretation of results and revisions to the manuscript. All authors read and approved the final manuscript.

## Conflict of Interest

The authors declare that the research was conducted in the absence of any commercial or financial relationships that could be construed as a potential conflict of interest.

The handling Editor declared a past co-authorship with one of the authors, NS.
