# Supplementary material for: Epigenetic Footprints of CRISPR/Cas9-Mediated Genome Editing in Plants
Source: Front Plant Sci. 2020 Jan 31;10:1720. doi: 10.3389/fpls.2019.01720 (PMC7026911; doi:10.3389/fpls.2019.01720)
Supplement: Supplementary file 1 [file DataSheet_1.docx]

**Table S1.** List of primers used in this study.

| **Primer name** | **Primer sequence (5’ – 3’)** |  |  |  |  |
| --- | --- | --- | --- | --- | --- |
| **Primers to confirm the presence of Cas9 gene in transgenic lines** | | | | |  |
| zCas9-F | CGGCCTCGATATTGGGACTAACTCT | | |  |  |
| zCas9-F | CTTATCTGTGGAGTCCACGAGCTTC | | |  |  |
| **Primers to amplify the fragment flanking target site for AT1G72350** | | | | |  |
| MP5-F | CTTCCAAACCCGTCCTGTAA | | |  |  |
| MP5-R | TGGTATTAAATGCGCGTCTC | | |  |  |
| **Primers to amplify the fragment flanking target site for AT1G09970** | | | | |  |
| MP8-F | ACGAGTTTGGCAGGACGTTA | | |  |  |
| MP8-R | CTTAAGCTTTGCCGCTTTGA | | |  |  |
| **Primers to amplify the fragment flanking target site for AT3G17320** | | | | |  |
| MP14-F | CCGAGACACACAAGAGGTGA | | |  |  |
| MP14-R | TTCGATCAAAAGACGGAACC | | |  |  |
| **Primers to amplify the fragment flanking target site for AT5G28770** | | | | |  |
| MP18-F | CCCCTTTCAAACGTGGAATA | | |  |  |
| MP18-R | GATTGGGCAGTCTTGCATTT | | |  |  |
| **Primers to make double strand gRNA fragment targeting AT1G72350** | | | | |  |
| gRNA1-F | ATTGAATACTGACTAATGAACCCG | | |  |  |
| gRNA1-R | AAACCGGGTTCATTAGTCAGTATT | | |  |  |
| **Primers to make double strand gRNA fragment targeting AT1G09970** | | | | |  |
| gRNA2-F | ATTGCTACACTACATGGTAGGCTT | | |  |  |
| gRNA2-R | AAACAAGCCTACCATGTAGTGTAG | | |  |  |
| **Primers to make double strand gRNA fragment targeting AT3G17320** | | | | |  |
| gRNA3-F | ATTGTTAAAGGTGGTACCAGCAGT | | |  |  |
| gRNA3-R | AAACACTGCTGGTACCACCTTTAA | | |  |  |
| **Primers to make double strand gRNA fragment targeting AT5G28770** | | | | |  |
| gRNA4-R | ATTGCCCTTTATGGTAGAGGACGT | | |  |  |
| gRNA4-R | AAACACGTCCTCTACCATAAAGGG | | |  |  |
| **Primers to amplify the template for in vitro sgRNAs synthesis** | | | | |  |
| T7_sgRNA-R | GCACCGACTCGGTGCCACTT | | |  |  |
| T7_MP5 (AT1G72350)-F | TAATACGACTCACTATAGGGAATACTGACTAATGAACCCG | | |  |  |
| T7_MP8 (AT1G09970)-F | TAATACGACTCACTATAGGGCTACACTACATGGTAGGCTT | | |  |  |
| T7_MP14 (AT3G17320)-F | TAATACGACTCACTATAGGGTTAAAGGTGGTACCAGCAGT | | |  |  |
| T7_MP18 (AT5G28770)-F | TAATACGACTCACTATAGGGCCCTTTATGGTAGAGGACGT | | |  |  |
| **Primers to amplify the fragment flanking potential off-target sites for AT1G72350** | | | | |  |
| MP5-off-F1 | AGCTGACCACTGCAACACAT | | |  |  |
| MP5-off-R1 | ATGGCGTTTGGTCTCATTTC | | |  |  |
| MP5-off-F2 | CCACTTTGGATTCCTTTTGC | | |  |  |
| MP5-off-R2 | ACCGCCTTAAAATTGAATCGAA | | |  |  |
| MP5-off-F3 | AGGAAGGCAGGTTGAAATCC | | |  |  |
| MP5-off-R3 | GGAACCACGTACCTCAGCAT | | |  |  |
| MP5-off-F4 | TCGTTTCTGTTCCGGTTTTC | | |  |  |
| MP5-off-R4 | CGCCTTAAAATTGAATCGAA | | |  |  |
| MP5-off-F5 | TGATTGAGGCCAATGTTTTG | | |  |  |
| MP5-off-R5&6 | CGGGTGTGGGTTTTAAAAAGT | | |  |  |
| MP5-off-F6 | TTGAGGGTTTTCACCAATCC | | |  |  |
| MP5-off-F7 | CAGTGTTAGACATTTAGTTGGATTTCA | | |  |  |
| MP5-off-R7 | GCCCATCAAAATTTGCCATA | | |  |  |
| MP5-off-F8 | GGTGTTAGACATTTAGTTGGATTTCA | | |  |  |
| MP5-off-R8 | TCCTCCCACCAACAACATTC | | |  |  |
| **Primers to amplify the fragment flanking potential off-target sites for AT1G09970** | | | | |  |
| MP8-off-F1 | CCAAGGAGATGACCCAGCTA | | |  |  |
| MP8-off-R1 | GGTCTCACAAGATGGAACTGG | | |  |  |
| MP8-off-F2 | ACTTGCTTTTCAGCCAAGGA | | |  |  |
| MP8-off-R2 | CCACGCCTGTACAAGAACAA | | |  |  |
| **Primers to amplify the fragment flanking potential off-target sites for AT3G17320** | | | | |  |
| MP14-off-F1 | ATCAATCGGGATTGGATCTG | | |  |  |
| MP14-off-R1 | AGTGCCATTGCCTTTGAAAC | | |  |  |
| MP14-off-F2 | GACGAACTTTGAGCCTCTGG | | |  |  |
| MP14-off-R2 | CATCCTCTTCCCAAGATCCA | | |  |  |
| MP14-off-F3 | AACGCTTTACGAAATTCCAG | | |  |  |
| MP14-off-R3 | AACCAAAACCAGCCAATATGA | | |  |  |
| MP14-off-F4 | ACCAGTTTTGGTCCAGGAAA | | |  |  |
| MP14-off-R4 | TGAGAACACCATCAGGAGCA | | |  |  |
| MP14-off-F5 | AGACACTGCTTTCACCACACA | | |  |  |
| MP14-off-R5 | GCCAAACATAGCTGTGATGC | | |  |  |
| MP14-off-F6 | ACCAAGATTGGCGAGAGATG | | |  |  |
| MP14-off-R6 | CTGAGAGTTCGCTGTTGTCG | | |  |  |
| **Primers for bisulfite genomic sequencing AT1G72350** | | | | |  |
| MP5_BS-F1 | AGATTTTGGGAAGATTGTTAAATGT | | |  |  |
| MP5_BS-R | TCTTTAATTAATTAACCACTCCACAAA | | |  |  |
| **Primers for bisulfite genomic sequencing AT1G09970** | | | | |  |
| MP8_BS-F | TATAAAATAAAAATATTTTATAGATAGATATAATATATAAATTTTTATATATATTAA | | |  |  |
| MP8_BS-R2 | CTTAAACAAAACTTAACTAAAATCATTATACCCCCAA | | |  |  |
| **Primers for bisulfite genomic sequencing AT3G17320** | | | | |  |
| MP14_BS-F | AAGGATGTYGTTAAGAAG | | |  |  |
| MP14_BS-R1 | AATTCTTTARATCCCTTTACTTTTAT | | |  |  |
| **Primers for bisulfite genomic sequencing AT5G28770** | | | | |  |
| MP18_BS-F | TTAATAATTTTTGTTTTYTTAATAATATTATTTATTTTTTTTATAAAATGGT | | |  |  |
| MP18_BS-R | CTTAACCCACCTTATCTCAATATCTTTCTTC | | |  |  |
| **Primers for bisulfite genomic sequencing lambda phage DNA** | | | | |  |
| Lambda_BS-F2 | TACAGAAAGACGGACGAAGG | | |  |  |
| Lambda_BS-R2 | TGGTGGGCGTTTTCATACAT | | |  |  |


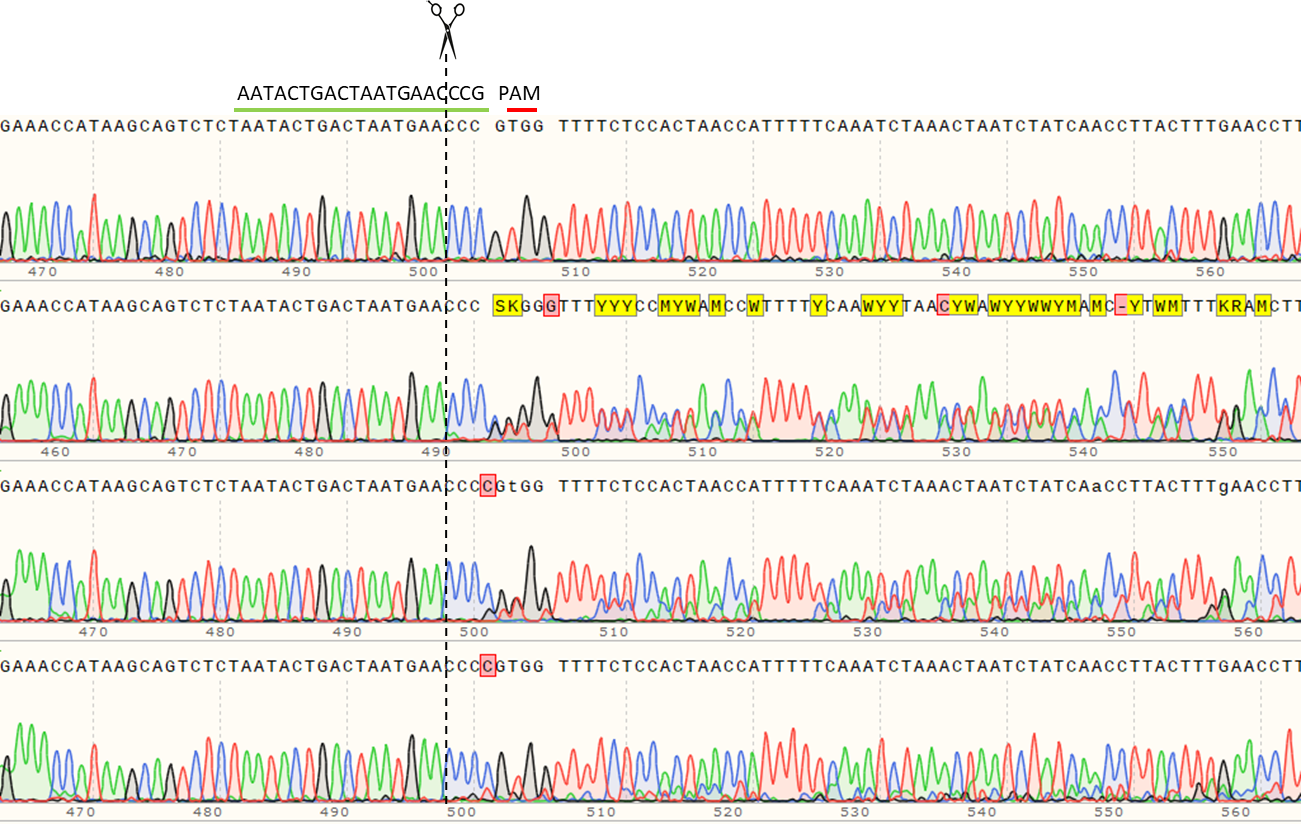


**Figure S1.** Genotyping of T_1_ plants targeting AT1G72350 gene. PCR amplicons flanking the target site were directly sequenced and then aligned. Top panel showed the sequencing chromatogram of wild-type and the below showed those of independent edited T_1_ plants. Multiple peaks of sequencing chromatogram in downstream from expected Cas9 cleavage site (3-nt upstream from PAM, marked with scissors and dot line) indicated InDel mutation was created.


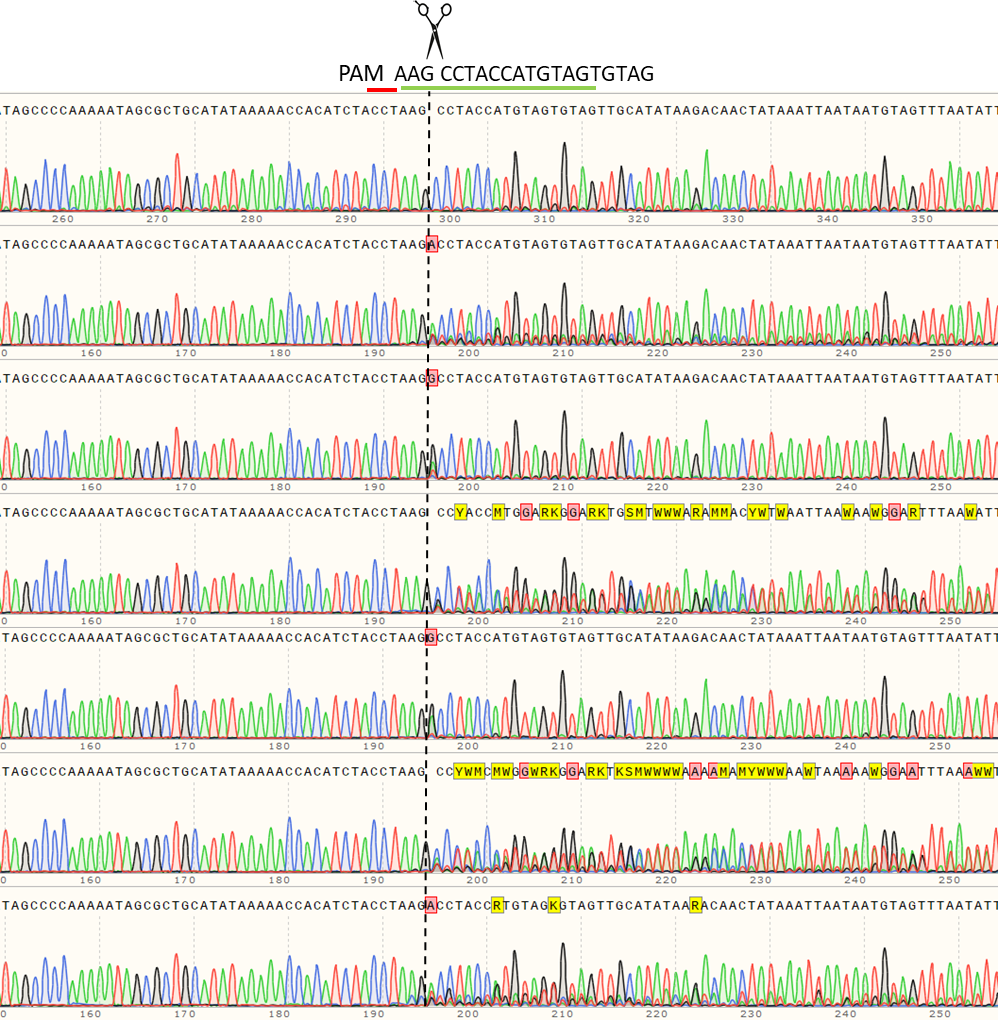


**Figure S2.** Genotyping of T_1_ plants targeting AT1G09970 gene. PCR amplicons flanking the target site were directly sequenced and then aligned. Top panel showed the sequencing chromatogram of wild-type and the below showed those of independent edited T_1_ plants. Multiple peaks of sequencing chromatogram in downstream from expected Cas9 cleavage site (3-nt upstream from PAM, marked with scissors and dot line) indicated InDel mutation was created.


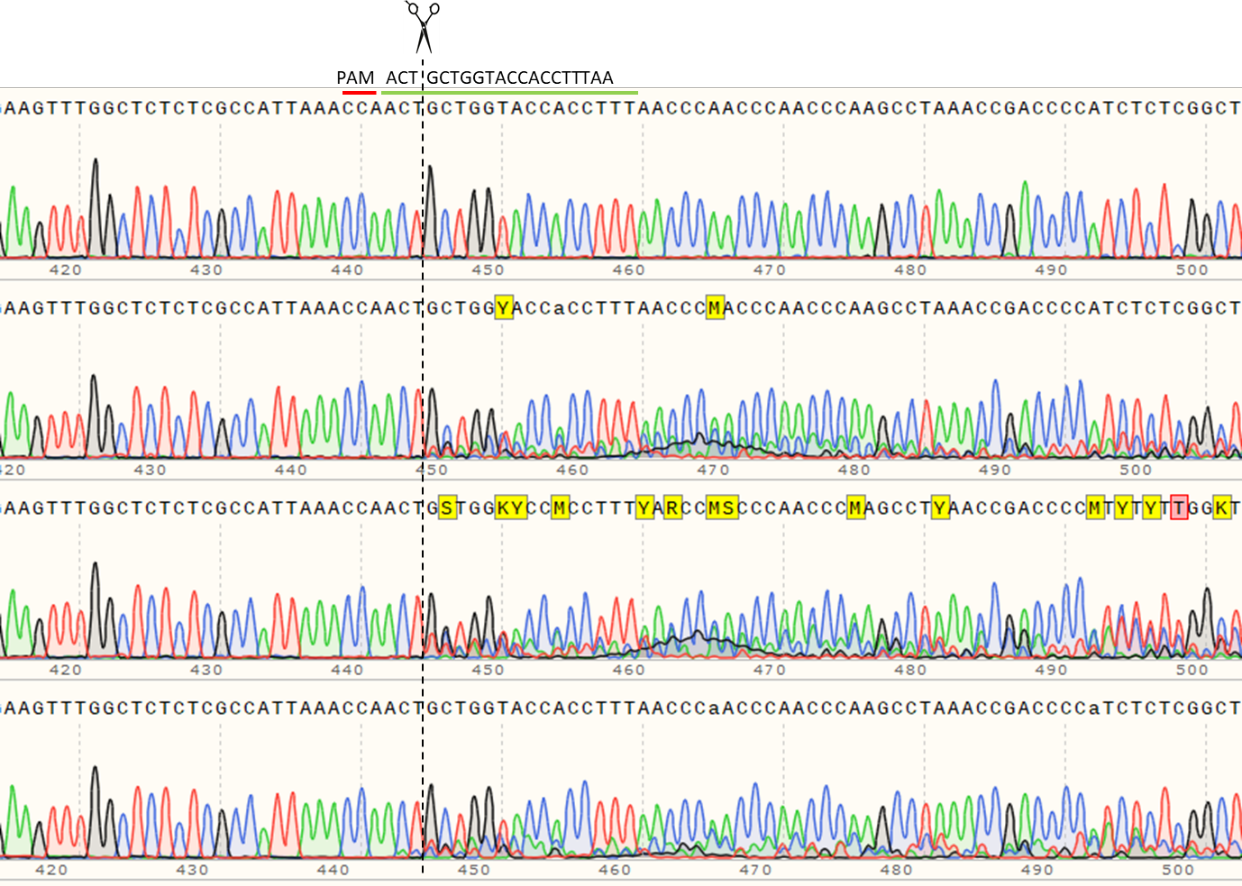


**Figure S3.** Genotyping of T_1_ plants targeting AT3G17320 gene. PCR amplicons flanking the target site were directly sequenced and then aligned. Top panel showed the sequencing chromatogram of wild-type and the below showed those of independent edited T_1_ plants. Multiple peaks of sequencing chromatogram in downstream from expected Cas9 cleavage site (3-nt upstream from PAM, marked with scissors and dot line) indicated InDel mutation was created.

**
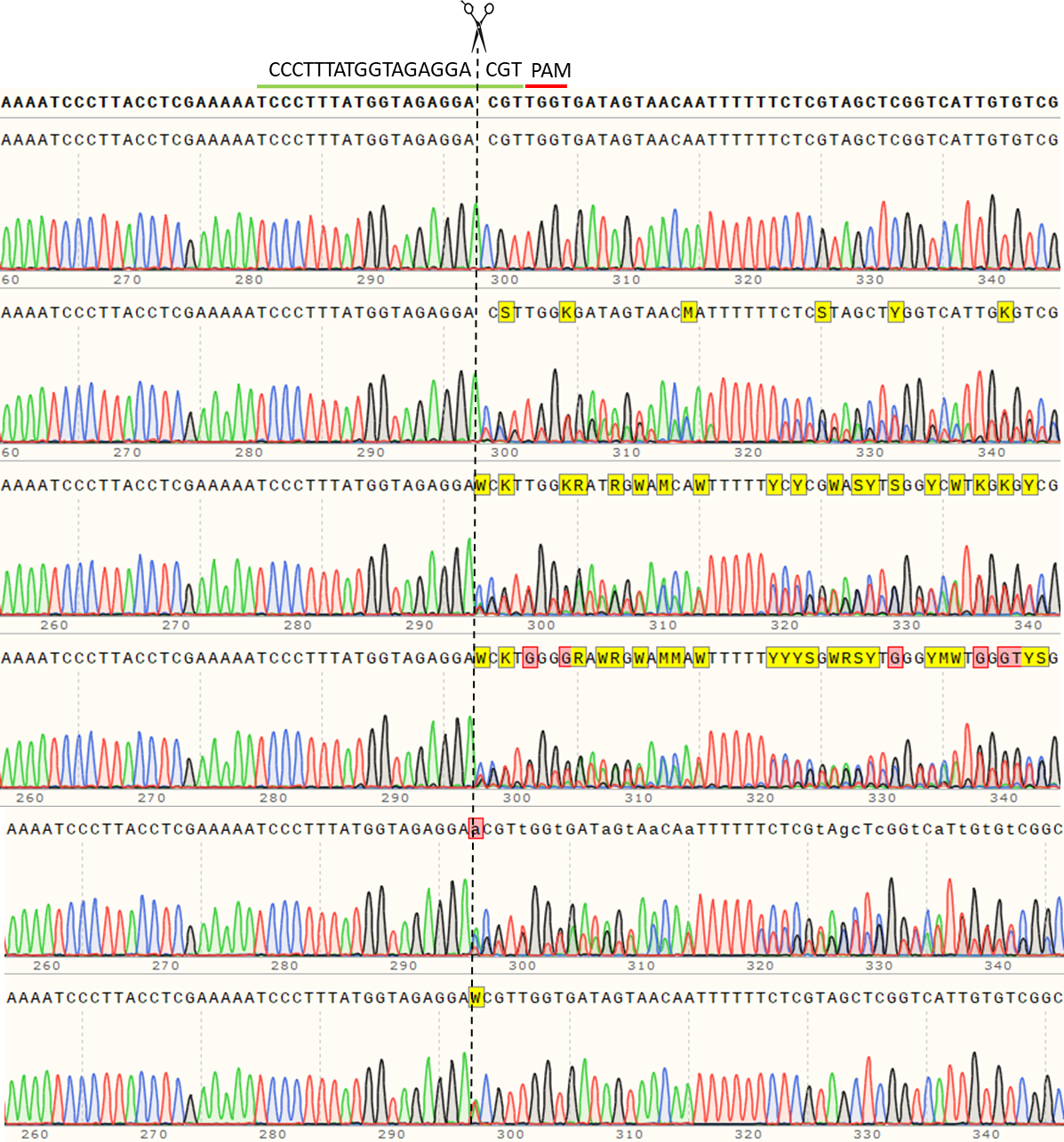
**

**Figure S4.** Genotyping of T_1_ plants targeting AT5G28770 gene. PCR amplicons flanking the target site were directly sequenced and then aligned. Top panel showed the sequencing chromatogram of wild-type and the below showed those of independent edited T_1_ plants. Multiple peaks of sequencing chromatogram in downstream from expected Cas9 cleavage site (3-nt upstream from PAM, marked with scissors and dot line) indicated InDel mutation was created.


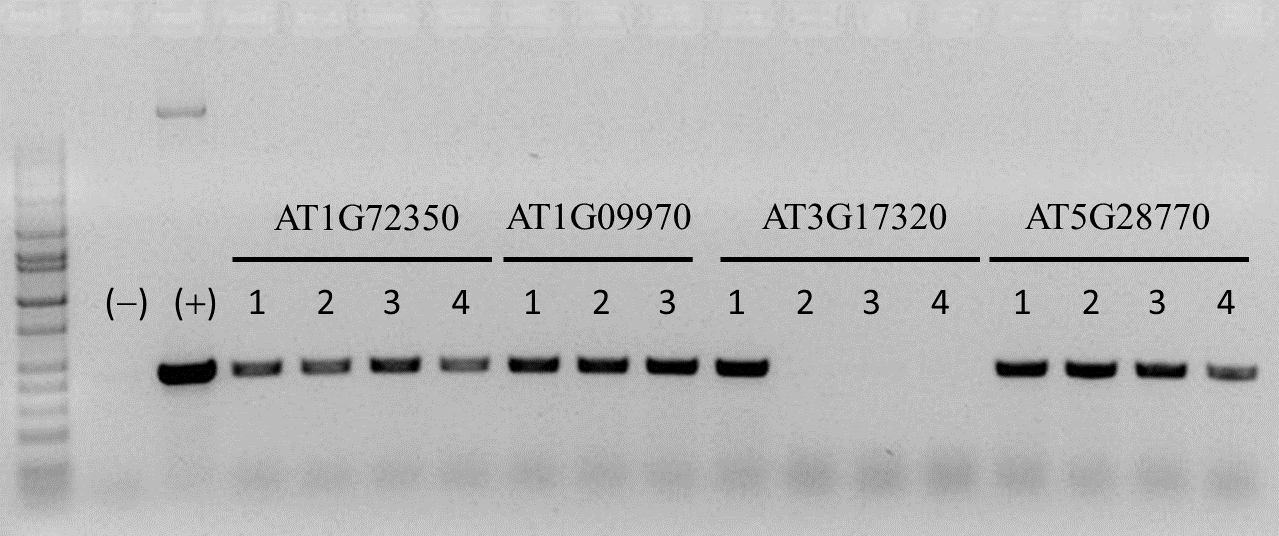


**Figure S5.** PCR confirmation of the presence of *Cas9* gene in independent edited T_2_ plants targeting AT1G72350, AT1G09970, AT3G17320, and AT5G28770. Most of the tested plants still contained *Cas9* gene, although escape of the gene was detected from three independent plants targeting AT3G17320 gene. (−), wild-type negative control; (+), pKSE401 plasmid positive control; 3-4 independent edited T_2_ lines of each target gene.

**
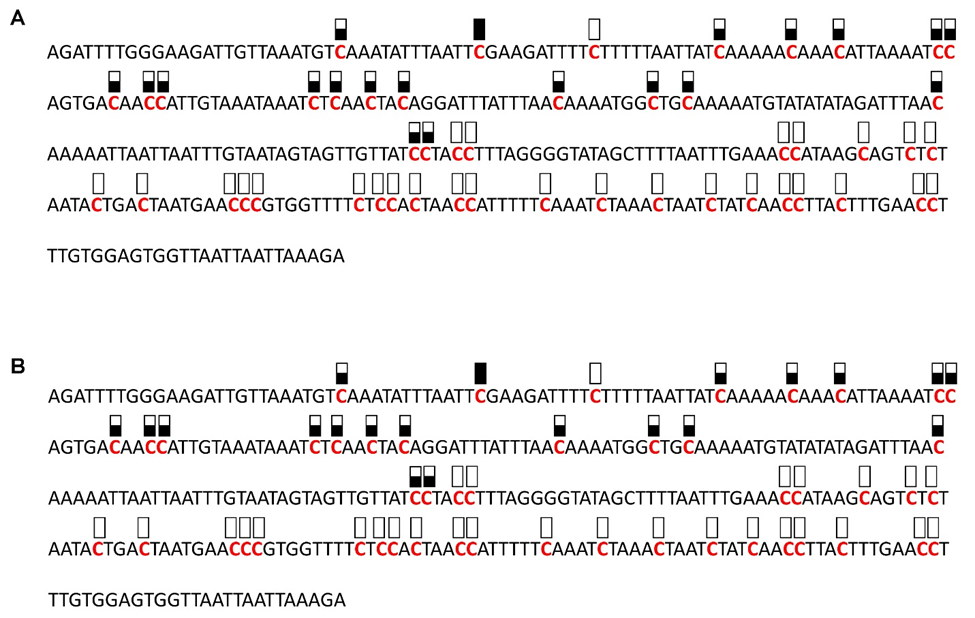
**

**Figure S6.** Pattern of methylation of the (AT1G72350) region in wild-type (A) and in the transgenic AT1G09970-edited (B) plants. Because the data were obtained by sequencing of independent pGEM-T colonies, an average level of methylation was determined for each cytosine. Solid boxes indicate that the cytosine at this position were methylated (>90 %), open boxes indicate that cytosine methylation was not detected (<10 %), and half-shaded boxes indicate that the cytosine was methylated at a range of 40-60 %. This figure presents results of 4 independent biological replicates.


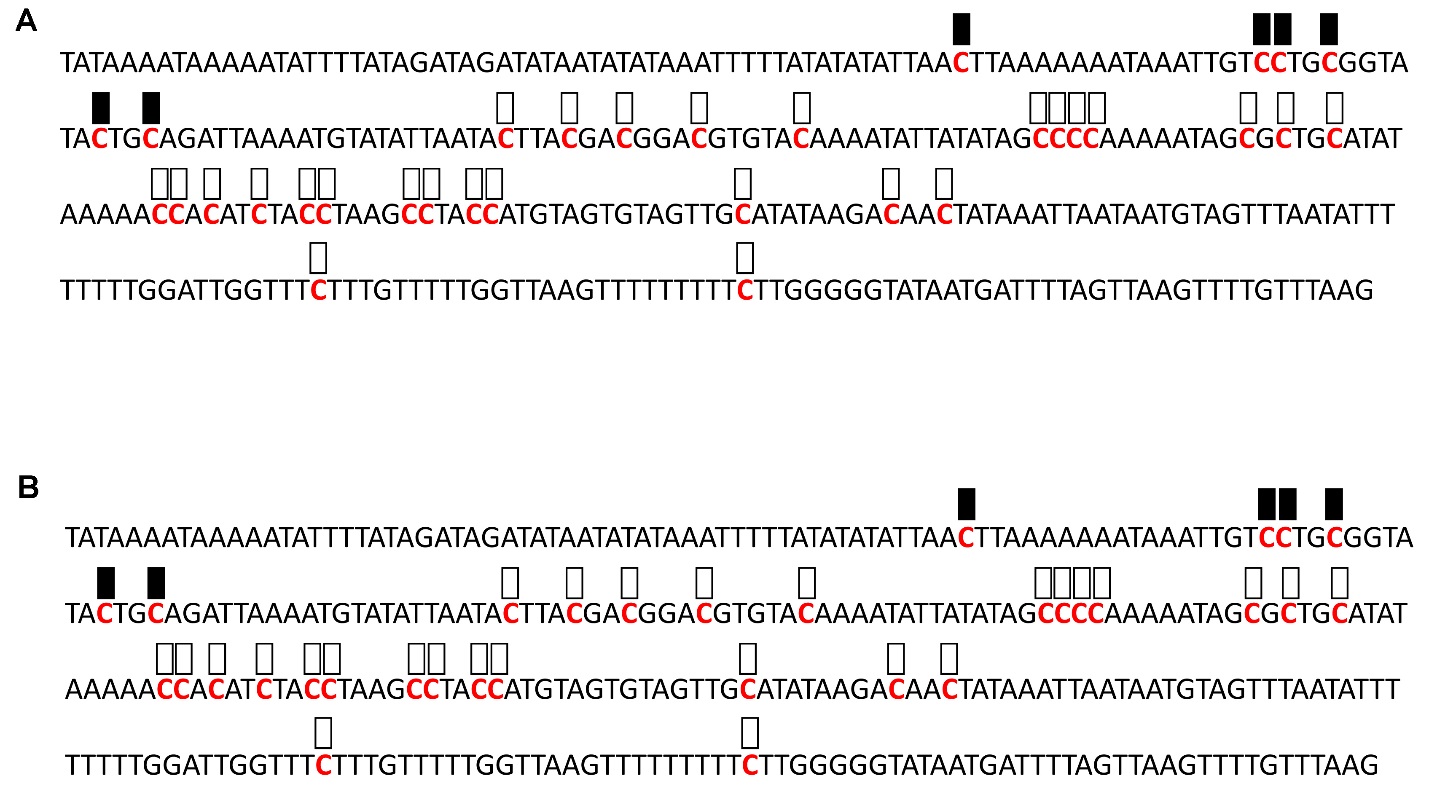


**Figure S7.** Pattern of methylation of the (AT1G09970) region in wild-type (A) and in the transgenic AT1G72350-edited (B) plants. Because the data were obtained by sequencing of independent pGEM-T colonies, an average level of methylation was determined for each cytosine. Solid boxes indicate that the cytosine at this position were methylated (>90 %), open boxes indicate that cytosine methylation was not detected (<10 %), and half-shaded boxes indicate that the cytosine was methylated at a range of 40-60 %. This figure presents results of 4 independent biological replicates.


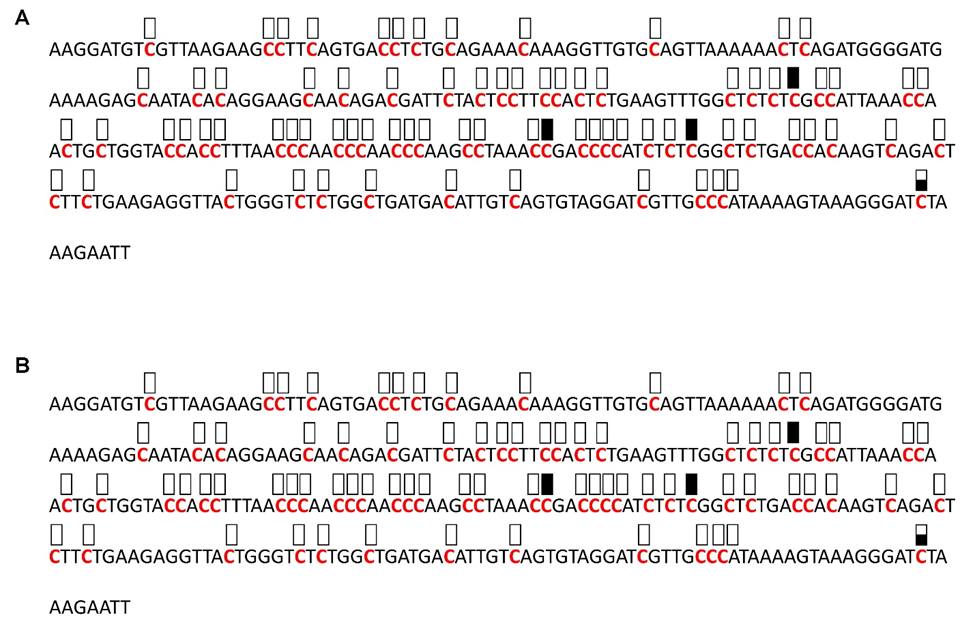


**Figure S8.** Pattern of methylation of the (AT3G17320) region in wild-type (A) and in the transgenic AT5G28770-edited (B) plants. Because the data were obtained by sequencing of independent pGEM-T colonies, an average level of methylation was determined for each cytosine. Solid boxes indicate that the cytosine at this position were methylated (>90 %), open boxes indicate that cytosine methylation was not detected (<10 %), and half-shaded boxes indicate that the cytosine was methylated at a range of 40-60 %. This figure presents results of 4 independent biological replicates.


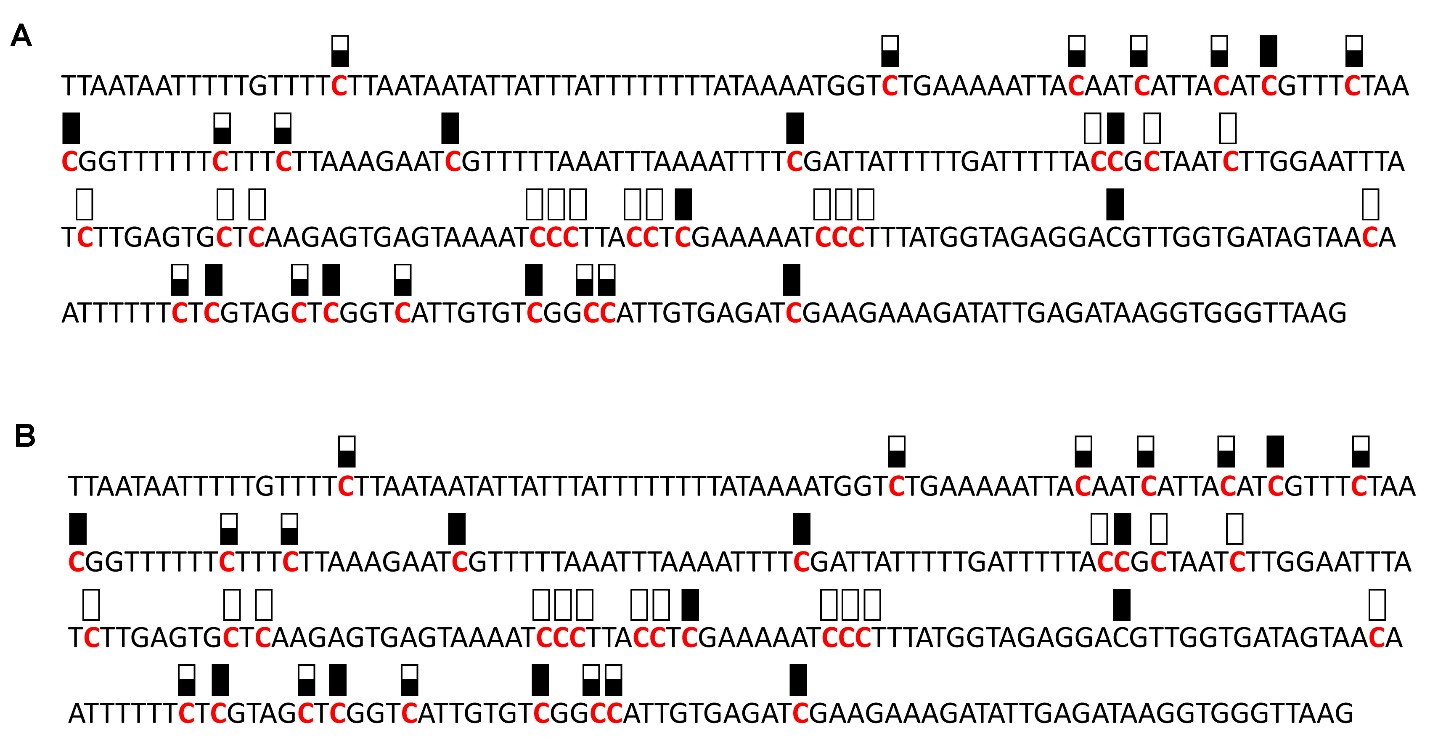


**Figure S9.** Pattern of methylation of the (AT5G28770) region in wild-type (A) and in the transgenic AT3G17320-edited (B) plants. Because the data were obtained by sequencing of independent pGEM-T colonies, an average level of methylation was determined for each cytosine. Solid boxes indicate that the cytosine at this position were methylated (>90 %), open boxes indicate that cytosine methylation was not detected (<10 %), and half-shaded boxes indicate that the cytosine was methylated at a range of 40-60 %. This figure presents results of 4 independent biological replicates.
